# Emotionally entwined narratives: a polyphonic trialogue on learning disability history research

**DOI:** 10.3389/fsoc.2025.1401521

**Published:** 2025-04-23

**Authors:** Owen Barden, Rhiannon Currie, Ian Davies, Helena Gunnarsdóttir, Jónína Hjartardóttir, Nathaniel Lawford, Jonathon Lyons, Sólveig Ólafsdóttir, Emily Oldnall, Sarah Oldnall, Guðrún Valgerður Stefánsdóttir, Amber Tahir, Samantha Taylor, Liz Tilley, Katrín Tryggvadóttir, Steven J. Walden, Heather Watts, Clare Wright, Christine Wright

**Affiliations:** ^1^TCPID, Trinity College Dublin, Dublin, Ireland; ^2^TRAC, University of South Wales, Wales, United Kingdom; ^3^SHLD, The Open University, Milton Keynes, United Kingdom; ^4^School of Education, University of Iceland, Reykjavik, Iceland; ^5^The Brain Charity, Liverpool, United Kingdom; ^6^Faculty of Life Sciences and Education, University of South Wales, Treforest, United Kingdom

**Keywords:** emotions, emotional community, learning disability studies, critical disability studies, inclusive research, autobiography, polyphonic conversation, activism

## Abstract

This paper offers a critical analysis of the concept of “emotional community” in the context of our research into histories of learning disability. Emotional communities are places where people feel, express and make sense of emotions. They help us to understand that emotions are something we experience socially and not just individually. The paper is presented in the form of a conversation between many researchers. This is what we mean by “polyphonic,” which translates as “many voices.” Some of us have learning disabilities, and some of us do not. Although there are many voices, the authors belong to teams who worked on three learning disability history projects. Each team comprises researchers and self-advocates with learning disabilities and academics without. We use the word “trialogue” to mean discussion involving the three teams. In the discussion, we first talk about what we mean by “emotional community.” Then we talk about the purpose of emotional communities, and their “light” (good) and “dark” (bad) aspects. We also talk about a process called “commoning,” which is working to understand what we have in common. This leads into a discussion of the ethics of emotional communities. We conclude by reflecting on some of the possibilities and problems we see with emotional communities.

## 1 Introduction

We start this trialogue in the middle, on a day in July 2023. By a trialogue, we simply mean an extended conversation between three teams of learning disability history researchers. On that day in July 2023, three teams of researchers who had been independently researching histories of learning disability^1^ met for the first time at a seminar to explore history and activism. We met at the Social History of Learning Disability (SHLD) Conference at The Open University, which has a 30-year history of showcasing research done by and with people with learning disabilities. One of the three teams was from The Open University’s SHLD group, which had been exploring the role of life stories in both illuminating and facilitating experiences of belonging, primarily through the story of SHLD co-chair Ian Davies. The second team had worked on a project officially called *Inside the History of Learning Disability*, although it came to be known affectionately as *The Antonia Project* because it centred on the life history of one woman with a learning disability, called Antonia Grandoni. This team included members from the Centre for Culture and Disability Studies (CCDS) at Liverpool Hope University, The Brain Charity in Liverpool, and the Teaching and Research Advisory Committee (TRAC) at the University of South Wales. The team researched Antonia’s life history after finding it in a book published in 1877 by Dr. William Ireland, who was considered at the time to be one of Britain’s foremost experts on what we now call learning disabilities. The third team were from the University of Iceland. Their project was called *Bibi in Berlin*, and was about the life history of one woman with a learning disability, called Bibi, who was brought up on an isolated farm in Iceland called Berlin. All three teams comprised researchers and self-advocates with learning disabilities and academics without. The seminar was organised as part of international network and engagement activities built into the *Bibi in Berlin* project. The Icelandic team has a long association with The Open University’s SHLD group, and members of the SHLD group acted as academic advisors on the Bibi project. The Icelandic team had also been greatly influenced by *The Antonia Project*’s approach and methodology, resulting in some similar findings. As such, we all stayed behind for a day after the main conference was over, and spent a morning together with the aim of sharing details of our respective projects, exploring synergies and connections, and directions for future research.

The contribution our article makes is twofold: conceptual and epistemological. The conceptual contribution comes from our exploration of one emotional community and consideration of its potential for learning disability activism. The epistemological contribution comes from bringing together and giving equal weight to our diverse etic and emic ways of knowing about learning disability. The article thus brings together experiences, insights and theory from disability studies and the sociology of emotions in a novel way. In making this contribution we submit that our disability studies orientation offers a useful lens for understanding the power and potential of emotional communities. This power and purpose relate not only to feeling and expressing emotions within our community, but also harnessing them in activism. As an emotional community, we are activists, involved in learning disability self-advocacy organisations including The Brain Charity, People First, and Throskahjálp. This, of course, returns us fittingly to the origins of disability studies and disability rights, which are rooted in activism: activism motivated by emotions such as anger, injustice, and hope. We think this rehabilitation of emotions is worthwhile (Barbalet, 2008) when the emotions of people with learning disabilities have long histories of being proscribed, monitored, regulated and pathologised.

### 1.1 The three stories

Ian Davies was born in England in 1955. He was sent to a residential special school as a young boy and then spent many years in learning disability day care services, interspersed with periods of employment and volunteering. In the early 1990s, Ian became a founding member of Northamptonshire People First, and subsequently gained a national and international profile as a leading self-advocate. In 2019 he collaborated with Liz Tilley to record his life story, which explored experiences of loss, relationships, challenges and achievements. Ian’s first experience of sharing his life story publicly in 2019 at an SHLD conference was an unexpectedly emotional experience—both for Ian, and for members of the audience. Later that year, Ian shared his life story in Japan as part of an international project to explore experiences of belonging for people with learning disabilities (Tilley, 2020). This included presenting to Japanese social work students who reported feeling very moved by his story. The project resulted in a manga version of Ian’s life story which he shared at our seminar in July 2023.

Both Antonia and Bibi spent much of their lives in institutions: Antonia in a hospital in Milan, between approximately 1840 and 1870, where she was subject to frequent visits and examinations by professors and doctors, which continued even after her death. Dr. Ireland compiled the various ‘expert’ reports into a case history to include in his book *On Idiocy and Imbecility* (Ireland, 1877). Bibi was institutionalised in a home for older people in Blönduós in 1958, following the death of her mother. This was not an uncommon experience for people with learning disabilities in the mid twentieth century. Bibi was only 31 at the time of her admission, and lived in the home for 17 years until she was supported to move into the community. After her death, the Icelandic researchers were gifted Bibi’s 145,000-word handwritten autobiography to review, and subsequently discovered Bibi’s diary and numerous poems that she had written. These texts, alongside other artefacts such as Bibi’s extensive doll collection, revealed a complex and insightful interior emotional life (Stefánsdóttir et al., 2025). Like Antonia, Bibi often seemed to crave emotional connections and caring human relationships. Analysis of Bibi’s autobiography led the team to theorise Bibi’s life in terms of the “emotional communities” she belonged to (Rosenwein, 2006).

### 1.2 How we have worked together

The concept of emotional community was a new one to the rest of us, and immediately of great interest. The call for papers for this special issue had come out just before we met, and as our conversations on that day came to a close, we agreed to co-author a paper on emotional communities and learning disability history as a way of continuing the work. This is why we say we are starting in the middle: the day we met represents a kind of starting point, but our trialogue reaches back into the past as we discuss the research projects that led up to the meeting, and forwards as we continued it in a series of meetings set up to facilitate the writing of this paper, and on into the future as we close the paper by considering some implications of our theorising of emotional communities in the context of international learning disability history research.

Previous work we have published has argued the case and paved the way for the contributions of co-researchers with learning disabilities to be rightfully accredited by publishers and the academy in the form of joint authorship (Barden et al., 2022; Tilley et al., 2021). Equality of authorship in this article reflects the collaborative and co-produced nature of our work, building on a growing movement that seeks to privilege ‘pluralistic ways of knowing’ (Durose et al., 2022). It also exemplifies our ongoing commitment to critique and challenge the ableist forms (and norms) of knowledge production and dissemination within academic publishing that threaten to both stifle and render invisible the critical contributions made by disabled colleagues. Inclusive research prioritizes co-creation, seeking to ensure that the voices and perspectives of people with learning disabilities are not only represented but actively shape the research process and its outcomes (Walmsley et al., 2018). Flexibility, responsiveness and acknowledgement of each person’s capabilities is key. In our field of research, authorship can never simply be about who physically writes the text, or who reads and comments on iterative drafts of an article. This version of authorship would soon become highly exclusionary. Instead, we argue that authorship can and should be a politicised (if contested) space in which we work carefully to identify alternative and creative mechanisms to facilitate people’s involvement in the publication process. It also involves articulation of the diverse and meaningful contributions (intellectual and experiential) made by authors with range of personal and professional backgrounds, and differing communication needs and preferences. Through listening to the voices of authorship in different ways that allowed those voices to be fully heard, we formulated our adaptive dialogic interpretative methodology.

In practical terms, this meant that our article came about primarily through a series of meetings in which personal and collective insights were generated, reflected upon and further interpreted, and ultimately written down. Some of these meetings involved representatives from each team sharing and reflecting on the emotions associated with learning disability history research and the nature of our own emotional community; these meetings were audio-recorded and transcribed. Other meetings involved each team reflecting separately on these issues at times and in places that worked well for them. These team reflections were then fed back to the wider group on Zoom calls and by email and subsequently embedded into this article as we commenced the writing process. Although the academic participants took on the bulk of ‘writing’ task (typing words onto the screen and providing some contextual content), we would have had little to write about were it not for those shared conversations. The result was described by one author as a kind of ‘inclusive and reflexive narrative’. Everybody who contributed to these discussions, and who wanted to be, is therefore named as an author on the paper.

The trialogue we present below is woven together into what we are calling an *emotionally entwined narrative* of learning disability history research. Quotations from our meetings and email conversations are presented in italics throughout the article to help distinguish individual reflections from our collective interpretations. Our meetings, and the writing of this article, have been something of an experiment in sharing memories and building an understanding of what we witnessed, and crucially *felt* on that day in July 2023. In doing so we attempted to activate ‘emotionally engaged’ methods for group analysis and interpretation (Thomson et al., 2023), working collaboratively and intuitively to unpack the emotional community that had been rendered visible during our seminar. Specifically, we were interested in which emotions were in play on that day, and why; how these emotions might point to areas of commonality between us; and the potential impacts of these emotions on future learning disability history research, particularly research which is animated by activist principles. In keeping with our inclusive ethos, we have tried to write the bulk of the article in the most accessible language we can, ensuring that the voices of all co-authors were captured and thus heard within that writing while maintaining the degree of criticality and rigour appropriate to an academic journal.

This paper therefore contributes a theorisation of the role and nature of emotions and emotional communities in learning disability history research. It sits at the intersection of history, sociology, disability studies and narrative research, although reviewing these bodies of work is beyond the scope of this paper. Although there is a growing literature on learning disability history, explicit discussions about the place of emotion within that history are rare—but see Rolph and Atkinson (2010) for a unique and important contribution. This is a branch of social history that has evolved since the 1990s in highly inclusive ways, developing research methods to proactively address archival silences and distortions, and to foreground the experiences of people with learning disabilities and their families (Atkinson and Walmsley, 2010). More recently we have seen a growing number of self-advocate historian activists leading their own heritage projects, exploring ways to use history for social change (Jarrett and Tilley, 2022). As such, it seems to us that this is an important moment in which to take stock of the emotional dimensions inherent in a field of scholarly inquiry that is both highly inclusive and often political and politicised.

It is well known that people with learning disabilities tend to have smaller social networks than the general population, often restricted to family, members of staff and friends/acquaintances made through services (Harrison et al., 2021). While social media has opened up opportunities for many disabled people to develop social connections across geographic boundaries (national and international), there are ongoing challenges regarding digital inclusion for people with learning disabilities (Chadwick et al., 2023). Opportunities for international travel appear to be limited for many people with learning disabilities (Sánchez-Padilla et al., 2024), and so our emotional community offered a unique space in which people could both expand their social networks through research endeavours, while sharing experiences that were intergenerational and geographically distinct. The day we met was itself very emotional. We talked a lot about the emotions we felt when doing research about the history of learning disability. These run the whole gamut from shock, disgust and outrage at the way people with learning disabilities have been, and continue to be treated; to defiance and pride; to taking delight in sharing stories and producing creative works to show what we have found and what we feel about it; to devotion to the cause of advocacy. Perhaps most affecting were shared moments of empathy couched in a developing sense of solidarity; a knowing glance was often enough to convey mutual understanding born of personal resonances with what was being discussed. A distinctive paradox of doing this kind of participatory research is how harrowing moments are juxtaposed with moments of laughter and joy. However, we seek to move the discussion beyond merely reporting the emotions people feel as they undertake this kind of research, to theorising both how research can bring us together as an emotional community, and how that emotional community might be harnessed in advocacy work. The article begins by defining the concept of emotional community, and critiquing it in the context of our learning disability history research. We then explore the emotions associated with our work, using metaphors of dark, light, dusk and dawn to describe how we are constantly moving between emotional states. The trialogue closes with some suggestions for how this theorisation might be developed in future work.

## 2 Emotional community

The concept of *emotional community* comes from Barbara Rosenwein, professor of mediaeval history at Loyola University, Chicago. In 2002, Rosenwein wrote a landmark article in the *American Historical Review* entitled “Worrying about Emotions in History” (Rosenwein, 2002). In it, she critiques grand historical narratives and scholars who argue that the emotional lives of people in mediaeval Europe were somehow more childish, simplistic, and coarse compared to later centuries. Now, it is fair to say that there is evidence to suggest that the expression of emotions—and the effects those expressions have—vary across cultures (Tarlow, 2012). Following from this, it is reasonable to assume the expression of emotions, and the effects of those expressions, will also vary across time. History, as the renowned learning disability scholar C.F. Goodey reminds us, is anthropology with time rather than place as the variable (Goodey, 2011). So, just as we cannot assume that labels like ‘idiot’ and ‘imbecile’ directly correspond with contemporary diagnostic labels like ‘moderate learning disability’, we cannot assume that historical emotions correspond exactly with emotions as we label and experience them today. Despite this caveat, we believe that the emotional community is a useful way of framing our learning disability history research, because it helps us make sense of what we do, how we do it and why we do it.

Rosenwein asserted that although we all possess an inherent, biological capacity to experience what we call emotions, how we label, express and react to emotions is not simply a personal matter, but shaped by culture and context. These cultures and contexts form emotional communities. Emotional communities are what give emotions names, values and respect; they are where we make sense of the emotions we feel, by sharing them with people who experience and evaluate them in similar ways. They are somewhat similar to speech communities, where people use language in specific ways in specific contexts (Matsumoto, 2013; Stefánsdóttir and Ólafsdóttir, 2021). Emotional communities therefore embody systems, cultures or conventions of feeling (Hochschild, 2008). A person can belong to multiple emotional communities simultaneously. Sola, who introduced us to this concept, uses the analogy of the public baths:

*Sola: I always use the analogy of going into an Icelandic swimming pool. You go to the showers, and you meet someone there and you're all naked and then you go out and you go to the hot tub and there's a political debate and then you go into the steam bath and everyone is just trying to survive the heat and then you go to the sauna, where you have very relaxed conversation, if you know someone there. And then you go for a swim. So you are in the same place the whole time, but you are in three or four different emotional communities while you are there. If you meet a best friend and you are going to have a very emotional talk, you go to some private area of the baths; in the political debate, you can choose from one hot tub or another by a political point of view.*

*Liz: That's really helpful. I like that analogy. Has anyone written about this from a disability studies perspective, or about how it might work across international contexts, as far as we know?*

*Sola: I don't know of anyone writing about this.*

*Owen: So Liz, were you thinking that this is how we might frame the contribution of this paper?*

*Liz: That's exactly what I had in mind. How we might expand the concept of emotional communities in some way to make it more inclusive, and to address that the literature to date has not necessarily attended to those issues around international context, different languages, but particularly, I think, issues around learning disability, and where some people may not use words to communicate.*

This, then, is the premise for the rest of the article. On the day we all met at the SHLD seminar, Sola defined emotional community and we came to realise that perhaps we were one, and had been one for some time, without knowing it. There was a sense that maybe coming together in the same room had somehow made our hitherto hidden emotional community manifest. But we could not be sure; we needed to think it through and doing so would involve reflecting on, critiquing, and elaborating on Rosenwein’s original concept. Our analysis of her concept forms the rest of the trialogue.

## 3 Emotions at play: the light, the dark and the liminal

Throughout our discussions we talked about what was emoted during our seminar and our reflections on those emotions in the months since. Our emotional responses were varied and specific, but there were commonalities too. Certain moments stood out as having prompted strong emotional reactions. One example was when Ian recounted to us all the first time he had told his life story to an audience, an event which he explained had been surprisingly challenging and which caused him to cry in the moment of telling of it. Afterwards, in meetings of the respective groups, some of the other researchers with learning disabilities told of how they had been particularly moved by Ian’s talk, because they felt they could empathise with him and his experiences, despite generational differences:

*Steve: Yeah, I've already had the first conversation, with Sam and Rhiannon from TRAC. And they brought up that they were quite emotionally affected by listening to Ian's experience because that was something that as younger people they haven't lived. But to listen to somebody who had been through it that really got to them, they said as much.*

*Helena: I was also thinking about that, because Ian's story impacted my group the most, because it was so accessible. And I think that was a key element.*

*Katrin: It has been really interesting but also sad. I have realized that things have changed even though it could be better. I felt it was sad and difficult to listen to Ian‘s story but I have also learned that I have in many ways a good life compared to Ian, Bíbí and Antonia. I think we got more understanding of each other and that we are strong and can do a lot of important things.*

*Jónina: I agree and I think also it is difficult to listen to people tell about difficult things in their life and I remember people with intellectual disabilities when I was growing up out in the country who were sent to Kópavogshæli (biggest institution in Iceland). I realise I was lucky, I was not sent to any institution. I have a family who cares for me and looks after me. I would like to know more about Antonia‘s life.*

This dawning realisation that through empathising with Antonia, Bibi, Ian and others we perhaps seemed to experience and express similar emotions about similar things when co-producing research on the history of learning disability was an early indicator that we might be an emotional community. Of course, membership is not as straightforward as everybody feeling identical emotions about the same time about the same things, because humans are complex beings who do not respond to things in identical ways. But what did seem to be important was how the emotional atmosphere promoted the experiencing and expressing of important emotions (de Rivera, 1992). There was a sense that we were in a safe space for showing and sharing these emotions. The importance of atmosphere and safe spaces was something that came through in the subsequent meetings we had in our teams:

*Helena: I talked to someone else from the Bibi project, and she is not used to talking about her feelings. She is afraid of talking about her feelings, because they are difficult. She remembered very hard feelings and complicated emotions, since she was a child. She also said that, during the Bibi project, she was able to talk about her feelings for the first time in her life. And to be able to sit with people who are also remembering complicated things, and, you know, sharing all these feelings that just brought up so many emotions for her. And I think maybe some of the others in the team.*

*Nathaniel: How incredible!*

This does indeed seem incredible—to become able for the first time in your life, when in your early 20s, to speak about the powerful and complicated emotions you feel. Clare and Christine are a mother and daughter who were part of The Brain Charity team on the Antonia Project, and described a similar liberating experience, this time not just about expressing emotions for the first time, but empathising for the first time and beginning to understand other people’s emotions. This is equally remarkable:

*Clare: It was the first time you opened up about your disability.*

*Christine: This is where I was going to check in. I wasn't able to speak much, because my epilepsy affected everything. However, I have always found it hard to appreciate emotions. I can't really read emotions at all. Therefore, to me, I became Antonia. Does that make sense?*

*Clare: Because she's had brain surgery - they removed the temporal lobe, the part responsible for emotion - she's sometimes not able to even show emotion or recognize other people’s. But through the work that we did, she certainly did. Yeah, and could talk about it in a way that she’s just never really talked about emotions before.*

*Owen: Oh, wow. That's pretty amazing. What do you think it was about that situation that helped that to happen? Was it reading Antonia story? Was it the people in the room? What?*

*Christine: First of all, reading her story, taking that it would have been me in that locked away situation. And sort of talking to this person I've never met before [Antonia], and saying what's your story? And so through that I sort of became Antonia but thought, I don't know if I'd like to be locked away. I am happy where I am now.*

Everyone seemed to agree that the positive atmosphere of a safe space with people with similar interests seemed to help people feel and express their emotions, contributing to these liberating and in some cases even revelatory experiences. Something important these discussions suggest to us—and there are many quotes we could use in addition to the ones above—is that within the safe space of our emotional community, people often felt able to think about and express complex and difficult emotions, and that in at least some cases this could be empowering. Feeling and expressing emotions within this safe space helped us to integrate as a community (Kemper, 2008). To extend our meteorological metaphor of light and dark, this integration helped foster a climate of solidarity and hope within our community, a climate reciprocally constituted by the emotional atmosphere of the day we met. Climates of solidarity—solidarity being a word which featured regularly in our trialogue—exist where people share a common cause and set of ideals (de Rivera, 1992). In our case, a belief in and commitment to disability justice. Climates of hope relate to people’s past and present levels of satisfaction and how satisfied they anticipate being in the future (op.cit). In our case, we may be less than satisfied with the present and the past, but we have hope that through our research and activism, we can change things for the better in the future, by changing the way people think about and respond to learning disability. We remember that disabled people came together in a movement to fight for social change not only because they were sad and angry, but because they had hope for a better future (Cosier and Ashby, 2016).

It is perhaps tempting to think that many or even most people might react to Ian, Antonia or Bibi’s stories in similar ways to us. But this is not necessarily the case. While people in our emotional community feel a sense of solidarity with each other, and respond to issues around learning disability in normative ways—being shocked and horrified by the same things, laughing at the same things, and so on—the long and often dismal histories of learning disability and learning-disabled people demonstrate amply that many people feel very differently about learning disability to us. One only has to think of the Do Not Resuscitate orders placed without consent on many learning disabled people during the Covid-19 pandemic (*People First*, including some of our co-researchers, rightly led vociferous opposition to this injustice in the United Kingdom); the higher mortality rates and poor healthcare in the years leading up to the pandemic; the litany of headline-making abuses in care homes and hospitals; or the moves to eradicate people with learning disabilities in Iceland, Nazi Germany and elsewhere to appreciate that many people are at best indifferent and at worst downright hostile towards people with learning disabilities (Barden, 2020a; Barden, 2020b; Barden et al., 2023).

We also think it is important to note here that emotional communities are not inherently good; that it is perfectly possible for people to belong to harmful emotional communities, where one finds oneself living at the mercy of that community’s emotional norms. This seemed to be the case not only for Antonia and Bibi—both of whom seemed to have craved affection and friendship during their lifetimes—but also for some of our learning-disabled researchers.

*Sola: With Bibi, we found that it’s two-way. So you can actually be forced into an emotional community that you don't like. And no, you cannot save yourself from it. That is something that I can feel once Bibi was inside the old people's home. Because she had a learning disability, people had power over her and she was forced into an emotional community that was not very good for her.*

*Owen: That hadn't even crossed my mind. I thought that emotional communities were things that you wanted to be involved in. I hadn't thought about people being part of emotional communities that they I didn't want to be in or that were, if you like, bad.*

Nathaniel, a researcher from the SHLD group who has autism,^2^ captured the range of emotions he experienced during our seminar, how they related to prior experiences within a harmful emotional community, and how he moved between these emotions, using metaphors of dark and light, rather than positive and negative or good and bad:

*Nathaniel: I find that my emotions on the whole seem to have a repeating narrative given meaning by lived experience and the order these emotions come in: shock and fear - being too young to experience institutions and scared of a repeat of history. Anger - people should be treated as people. Defiance - against this injustice. Pride - reflecting at the many things people have accomplished and the intrinsic worth of the human self. Devotion - a deep desire and a promise to serve humanity and prevent a repeat of history ever occurring again. I have found this narrative of emotion to be present within not just myself but many self-advocates and the researchers I have spoken with.*

*It is, I think, undeniably good to feel ‘bad’ emotions, for bad things have occurred which in an empathetic and emotionally intelligent community will inevitably bring up feelings that could be termed ‘bad’ or as I put it ‘dark emotions’. I think though a matter which is more complex and alluded to in this question, is by what metric do we state whether an emotion is ‘bad’ or ‘dark’? Do we need more explicit focus on the 'good' or ‘light’ emotions? And how do cultural norms and values affect our framework of emotions? To kick start this conversation off I will share how I measure whether an emotion is light or dark.*

*Light emotions are least likely to cause the individual to have a desire to cause physical injury to another individual. For example, happiness, joy, love are unlikely to be the direct instigators of aggressive action. Of course, these same emotions can be taken advantage of, and the person does not become invulnerable to doing harm when feeling these emotions. But the definition is not about protecting oneself from being taken advantage of, but rather protecting others from one’s own capacity for violence, and the possibility of that capacity being used with intent. Dark emotions are most likely to cause the individual to have a desire to cause physical injury to another individual. Note this does not mean that the results are bad but the emotion of itself is. For example, feeling anger at a carer abusing those they are meant to support could make one lash out with anger or fear, which potentially could stop the abuse from continuing. However, the intent was still to make another suffer and is therefore harmful. But we live in an imperfect world so it is a sad truth these moral compromises are sometimes necessary, but still constitute a failing in the ethical sense even if tactically there was little or no choice.*

Several other researchers within the group also commented on the importance of experiencing ‘dark’ emotions. What all this seems to suggest to us is that within our emotional community, as we move towards a common understanding of the histories and of each other, we are constantly cycling between the light and the dark (Edensor, 2015). We frequently find ourselves in the liminal spaces of dawn and dusk, not just in the overwhelming brightness of midday or the total blackness of midnight. It is this coming together to experience and make sense of a range of emotions through storytelling that defines us as an emotional community (Lemmelijn, 2012; Prendergast, 2022). And it is the emotions that make learning disability history research what it is.

## 4 Commoning

Our sense was thus that we did belong to an emotional community, one which allowed us to experience a wide range of emotions, and through doing so a sense of solidarity. Yet we wanted to test this hypothesis:

*Liz: Is it possible that we could have all of our conversations and conclude that perhaps we didn't form an emotional community? Given what we’ve said about people responding differently to events, like the day we all met, how can we be sure we belong to the same emotional community?*

*Owen: I think it’s a fair question, Liz - how do we know that we're part of an emotional community, other than just asserting that we are? How would we know if we weren't? This has got me thinking about what community is in general. If you set aside the emotional bit, I think it's a group of people having a common purpose. People have different roles and responsibilities within a community, but I think we have a shared purpose in the kind of research that we're undertaking and why we do it. And that brings me to one thing that has popped into my head while we've been talking. I've been using the work of Tim Ingold recently to think about learning disability. He's an anthropologist. He talks about the etymology of community. So, the ‘com-’ part is coming together. But the ‘-munity’ is munificence, like gifts. In other words, a community is where everyone has their gifts to give. That's what makes a community, the idea that everyone's got their gifts to give. But what happens when people struggle to give their gifts, because of the language barrier, or what have you?*

*Liz: That's really interesting. I really love that idea of communities as the giving of gifts, and then thinking about how people can be enabled to give their gifts, to be able to participate. That's quite a new concept for me to think of it like that, but it actually goes to the heart of so much of our thinking in disability studies, doesn’t it, that people are prevented at every turn from being able to give the gifts they have.*

*Owen: Another thing Tim Ingold talks about is ‘the commons’. Having something in common. But he actually talks about the process of commoning. This means continually creating things that you have in common rather than trying to assume that everyone's the same to begin with. So commoning is a process that we all undergo together. And it's toward creating a new sense of commonality rather than trying to work out what we had in common before we all started. And I think this notion of commoning might help define us as a community.*

*Liz: I wonder whether that process of commoning is enhanced by the display of emotions. Does expressing emotions enhance people acknowledging or realising that they have things in common? Because there was that realisation that all of our lives are so different … Different cultures, men, women, different ages, disabilities, but actually there are things that we share, whether it's values or experiences. Or is it the other way around - did we sense that day that we were a group moving towards commonality, and that is what provoked strong emotional responses?*

*Steve: It could be a reciprocal driver. It could be a case of emotional connection driving commoning, and commoning driving emotional connection. Maybe that intersection can't be broken. Maybe it shouldn't be.*

Our sense, then, is that our emotional community is a place where commoning happens (Ingold, 2018). Through commoning, we constantly enhance our understanding of each other, the gifts each of us can offer, and what we have in common. Our commoning is thus an expression of the appreciation of diversity that is characteristic of climates of solidarity (de Rivera, 1992). This process of commoning within an emotional community feels as though it is reciprocally driven by experiencing and expressing strong emotions (Collins, 2008).

## 5 Emotional community and ethics

Something important that we felt emerged from the day we met at the SHLD seminar was our sense of moral obligations to attend to, engage with and negotiate our own emotions, and to help others do the same (Shanks, 2022), because learning disability history, policies and practices can weigh heavily on all of us at times. As our discussions after the seminar progressed, we recognised that some emotions are generated by the ethical obligations that drive many of our shared research endeavours:

*Sola: You are going to have more cultural capital. And that is something I think it's something that I am a little bit afraid of, because bringing people into the community brings responsibilities with it.*

*Steve: That really resonates with me, because I've literally got that happening now, this week. I'll be seeing Owen on Wednesday because we're coming up to Liverpool for a conference. Samantha and B*^3^ *are coming with me. Samantha has been working with us for quite some time on a number of research projects. So she's used to this. But B, who is her partner, is new to it. But there's been this transfer of cultural capital, he got interested by being Sam's partner, and became more interested and decided to be part of this. So he's coming up just to see how this works. And moving more towards being a participant in research. But he's been chatting to me as well. And I'm feeling that same weight of responsibility.*

*Liz: That is a really interesting dilemma. And it connects, I think, to the discussions we were having last time around the ethics around a lot of this work, because I think there is a sense of responsibility amongst all of us. There is a sense I think when you've had an experience that was quite heightened emotionally, you do want to find ways that you can sustain that, it genuinely does feel like an ethical obligation, actually. But we're also dealing with the imperfect nature of the institutions in which we work. And that is not always that easy to do. I did feel that responsibility after our meeting last July, it provoked excitement but also anxiety, which is one reason why I'm so pleased we're able to do this paper because it gives us a mechanism to carry these conversations on.*

What this conversation highlights to us is the ethical obligations we feel in belonging to our emotional community; the need to continue the work in order to honour the gifts that people give to the community, and to carry the work of the community through to our activism. We conclude our analysis by discussing problems and possibilities we see for our emotional community, beginning with a consideration of potential purposes.

## 6 Discussion: possibilities and problems

We have started to ponder what our emotional community might *do*, beyond offering a closed space for commoning and mutual respect (Helm, 2014). One possibility is to harness it in advocacy and activism. Much learning disability research is motivated not just by a desire to find things out, but to amplify the voices of learning-disabled people in arguing for positive change which moves us towards a more inclusive and equitable society. This, as we said at the outset, returns us fittingly to the activist roots of disability studies. This is important when leading lights in the field have criticised much research for straying too far from its original path of enacting meaningful change (Barnes, 2022). One important aspect of such advocacy work is very basic yet also extremely difficult: getting people to care about people with learning disabilities, and learning disability histories. Anecdotal evidence suggests that our emotional community has the power to do just that. For example, Sola can tell a story about how on a visit to Japan, a colleague’s recounting of Bibi’s life history had such an impact that it reduced one of the host professors to tears. This is perhaps a tentative first step towards positive change; a way to make things happen. If you can get people to care about something so much that they cry, perhaps you have already made an important change that influences them to think and act differently in the future. How we might harness the emotional power of our community is something that we will keep at the forefront of our minds as we carry this work into the future in the United Kingdom and Iceland.

Our community has helped remind us that history is comprised of events that happened to people like us, and not just of things written into books. This understanding appears to translate across borders of both nations and age, and thus points to our common humanity. We argue that it is time for us to become more emotionally curious and to consider how a ‘willingness to follow feelings’ might ‘incite an analytic process that involves connecting individual stories to collective endeavours, social resistance and social research’ (Thomson et al., 2023, p. 14). Through the production of this article and the multiple conversations that informed it, we talked ourselves in, and out, and back into an emotional community. Our mutual research interests, underpinned by shared ethical and political concerns and combined with our own subjective and emotionally driven experiences of doing research in this field, persuaded us that we were actively engaged in a process of commoning that was coherent enough to constitute an emotional community. This realisation in many ways raised more questions than it answered. How might the concept of emotional communities, so heavily dependent in its focus on oral and textual capabilities, be further democratised to include people who may not use words to communicate? Given the ineffability of emotions—the challenges of expressing emotions even in your native language—how might emotional communities navigate the complexities that arise when we are commoning cross-culturally, and in different languages (Kahl, 2019)? We, for example, have speakers of English and speakers of Icelandic, but none of the native English-speakers speak Icelandic, and only a few of the Icelanders speak English. How can we be sure that we are talking about the same emotions? That we have found ways for everyone to give—and receive—their gifts (Martinez et al., 2016; Ferrari et al., 2022; Gaya-Morey, 2024)? What is the role of body language and facial expression, which one autistic member in our trialogue suggested some neurodivergent people may find hard to read? Having identified that we are an emotional community, what do we do next? What might this realisation animate in our practices, our priorities and our plans? Our ongoing discussions following the seminar have clarified that we all believe the research we engage in to be inherently emotional. We are all open to further analyses that explicitly foreground the layers of emotion that arise in, between and across different temporal moments in learning disability history and biographical research, and our own positions within those analyses. Perhaps most significantly, we are interested in what these emotions might provoke, and how they can be used productively to address the historic inequalities and slow violence facing people with learning disabilities across our respective contexts (Nixon, 2011; Mills and Pring, 2024; Stefánsdóttir et al., 2025). At our seminar there were tears, laughter, empathy, insight, resonance and solidarity. We look forward to the evolution of our emotional community and the directions in which it may take us.

## Footnotes

^1^‘Intellectual’ and ‘Cognitive’ Disability are terms referring to impairment of intellectual ability causing difficulty with learning and everyday activities. In the United Kingdom, this term is interchangeable with the term ‘Learning Disability’. A ‘Learning Disability’ in other parts of the world, such as the United States, refers to what is termed in the United Kingdom ‘Specific Learning Difficulty’ which instead refers to diagnostic labels such as Dyslexia and Dyscalculia.

^2^This is Nathaniel’s preferred phrasing; we acknowledge that some people prefer alternatives.

^3^Unlike Samantha, B is not a co-author and so has been anonymised.
